# Metabolic and skeletal muscle dysfunctions correlate with fatigue behavior in an animal model of multiple sclerosis

**DOI:** 10.3389/fphys.2026.1892106

**Published:** 2026-08-11

**Authors:** Kody M. Moore, Marcella Whetsell, Lei Wang, Ethan M. Meadows, Amanda Stewart, Alan D. Mizener, Stuart A. Clayton, Lauren E. Rentz, Christa Lilly, John M. Hollander, Gangqing Hu, Emidio E. Pistilli, Edwin C.K. Wan

**Affiliations:** 1 Department of Microbiology, Immunology, and Cell Biology, West Virginia University School of Medicine, Morgantown, WV, United States; 2 Division of Exercise Physiology, Department of Human Performance, West Virginia University School of Medicine, Morgantown, WV, United States; 3 Mitochondria, Metabolism & Bioenergetics Working Group, West Virginia University School of Medicine, Morgantown, WV, United States; 4 Animal Models and Imaging Facility, West Virginia University School of Medicine, Morgantown, WV, United States; 5 WVU Cancer Institute, West Virginia University School of Medicine, Morgantown, WV, United States; 6 Department of Epidemiology and Biostatistics, West Virginia University School of Public Health, Morgantown, WV, United States; 7 Department of Physiology, Pharmacology & Toxicology, West Virginia University School of Medicine, Morgantown, WV, United States; 8 Department of Dermatology, West Virginia University School of Medicine, Morgantown, WV, United States; 9 Department of Neuroscience and the Rockefeller Neuroscience Institute, West Virginia University School of Medicine, Morgantown, WV, United States

**Keywords:** EAE, fatigue, metabolism, multiple sclerosis, skeletal muscle

## Abstract

Multiple sclerosis associated fatigue (MS fatigue) is one of the most common and troubling symptoms experienced by people with MS, but the cause of MS fatigue remains unknown. The nature of cognitive and physical exhaustion suggests that MS fatigue can be a metabolic disease that impacts energy demanding processes such as neuronal activity and skeletal muscle contraction. In this study, we investigated how autoimmune-mediated inflammation causes skeletal muscle atrophy and metabolic dysfunctions, which may contribute to MS fatigue. Using a relapsing-remitting model of experimental autoimmune encephalomyelitis (EAE), we found that disease manifestations substantially altered the metabolic activities of the mice over time. EAE decreased muscle mass and force production in both fast extensor digitorum longus (EDL) and slow soleus muscles but only increased the rate of fatigue in the EDL muscle. Gene expression analysis of the EDL muscle shows that genes related to cellular respiration and mitochondrial functions were significantly downregulated when EAE manifestations were the most severe, but the phenotype was reversed during disease remission. In addition, EAE-induced mice showed fatigue behavior during a maximal treadmill test. Surprisingly, this fatigue behavior was also observed with mice immunized with a CNS non-specific antigen, suggesting there are contributions from central nervous system-independent immune activation. To our knowledge, this is the first report to characterize skeletal muscle properties and fatigue behavior in mice following EAE induction.

## Introduction

Multiple sclerosis associated fatigue (MS fatigue) is an “invisible” symptom without a clear clinical definition, so patients often find it difficult to explain their fatigue to others. MS fatigue is sometimes described by patients as general weakness or lack of energy, but for some patients it may be a complete mental and physical exhaustion, known as “lassitude”. MS fatigue can occur suddenly without reason, lasting for a very long time, and cannot be resolved even after rest. Some patients experience chronic MS fatigue, meaning that the symptoms are consistently present even when resting. Medications prescribed for MS fatigue are stimulants of the central nervous system (CNS) that do not resolve the root of the problem. Thus, we sought to elucidate mechanisms by which MS causes fatigue.

Primary causes of MS fatigue such as brain atrophy, axonal loss, and immune activation are proposed but their correlations are more suggestive than definitive (Chaudhuri and Behan, 2004; Braley and Chervin, 2010). Fatigue tends to be more common in patients with progressive disease, but there is no definite correlation between fatigue and MRI findings on total lesion load, number of enhancing lesions, or whole brain atrophy (Bakshi, 2003). Some studies suggest that cortical atrophy may be correlated with MS fatigue (Cantor, 2010; Pellicano et al., 2010), but a causal relationship has not been established. Importantly, cortical atrophy cannot explain why over 80% of MS patients, including those with predominantly white matter lesions, suffer from fatigue. Similarly, secondary causes such as depression, medications, and sleeping disorders cannot fully explain the prevalence of MS fatigue. Importantly, there is a lack of research investigating possible CNS-independent mechanisms on MS fatigue.

Metabolic and mitochondrial dysfunction can contribute to both MS and fatigue. The rate of oxygen consumption, oxidative phosphorylation, and ATP generation by mitochondria directly impact many cellular processes. Neuronal activity and skeletal muscle contraction both require a high demand of ATP. Several studies have shown that functional axonal mitochondria are essential to maintain the integrity of axonal structure (Waxman, 2006; Tsutsui and Stys, 2013). In demyelinated axons, mitochondria accumulate to meet a higher demand of energy imposed by the loss of myelin (Mahad et al., 2009; Kiryu-Seo et al., 2010). Importantly, several post-mortem studies reported mitochondrial abnormalities within neurons (Dutta et al., 2006; Broadwater et al., 2011; Campbell et al., 2011; Witte et al., 2013). The cause of mitochondrial dysfunction in neurons is not known, but it could be caused by immune activation and inflammation. Similarly, immune activation could directly cause muscle fatigue. Using clinical samples and a xenograft mouse model of breast cancer-induced muscle fatigue, we demonstrated that peripheral factors produced by cancer cells can remotely impact metabolic and mitochondrial activity of skeletal muscle, leading to muscle fatigue (Wilson et al., 2019, 2021). Although the cancer-induced fatigue and MS fatigue may be caused by different mechanisms, our studies suggest that a CNS-independent mechanism that contributes to MS fatigue may exist. In this study, we used a relapse-remitting MS (RRMS) model of experimental autoimmune encephalomyelitis (EAE) to address our central hypothesis that the autoimmune-mediated inflammation causes skeletal muscle atrophy and metabolic dysfunctions, thereby contributing to fatigue-like behavior in mice. This work would challenge the current dogma that chronic MS fatigue is attributed primarily to CNS pathology.

## Methods

### Mice

Female SJL/J mice (JAX 000686) were purchased from The Jackson Laboratory. The mice were housed under specific-pathogen-free facilities at West Virginia University Health Sciences Center. All studies were conducted in accordance with West Virginia University Animal Care and Use Committee guidelines.

### EAE induction and assessments

Female SJL/J mice were immunized subcutaneously with 100 μg [Ser^140^]-PLP_139–151_ emulsified in 200 μl complete Freund’s adjuvant (CFA) (Hooke Laboratories EK-0120) into the upper and lower flank. Control groups included mice that were not immunized or immunized with 200 μg ovalbumin (OVA) emulsified in 200 μl CFA (Hooke Laboratories EK-0301). Clinical scores were measured daily based on the extent of ascending paralysis. A five-point scale was utilized: 0.5: the tip of the tail loses strength; 1: limp tail; 1.5: limp tail with disrupted gait; 2: inability to maintain hind limb separation upon suspension, one hind limb dragging; 2.5: complete paralysis of one hind limb, with or without dragging of another hind limb; 3: full paralysis of both hind limbs; 3.5: complete hind limb paralysis with partial fore limb paralysis; 4: complete hind and fore limb paralysis; 4.5: complete paralysis, the animal is not alert; 5: moribund. Clinical remissions and relapses were determined by a full 1-point change in clinical disease score. Full remissions were identified by animals maintaining a score of 0 for more than 1 day. By endpoint, the majority of animals present with no observable clinical disability.

### Animal metabolic assessments

Immediately following EAE induction, the mice were individually housed in the Comprehensive Lab Animal Monitoring System (CLAMS Model 2018, Columbus Instruments) for 40 days with continuous monitoring using Oxymax version 5.68 (Columbus Instruments). Food and water were added *ad libitum* and cage internal environments were kept consistent (23°C, 800.98mmHg, O_2_ in ≥20.58%, 12Hr day/night light cycle). At the beginning of each run as well as every 7-days, the instrument was first purged with nitrogen gas and subsequently recalibrated with span gas (20.85% O_2_, 5% CO_2_, and 74.15% N_2_, Airgas 40-4000617690-1). A Paramax 101 oxygen sensor and automatic carbon dioxide sensor (Columbus Instruments) was utilized to take environmental measurements. Any readings that deviated from standard environmental conditions resulted in that run being discarded and subsequently supplemented with additional biological replicates. Disease scoring and assessment of animals was performed off-cycle of cage sampling as to not affect internal environments. The volume of oxygen consumed by animals (vO_2_) and carbon dioxide produced by animals (vCO_2_) within the environment was recorded. The respiratory exchange ratio (RER) was calculated as vCO_2_/vO_2._ In brief, RER is used to determine substrate usage for metabolism and determines if animals are in a state of aerobic or anerobic state of metabolism. Key RER values are as followed: near or below a value of 0.70 indicates primary fat usage and is considered to be a state of starvation and is also correlated with long-term high intensity activity, values at or above 0.85 indicates a balanced usage of both carbohydrates and fats most commonly associated with a steady resting state, values near or at 1.0 indicates primary use of carbohydrates most commonly associated with activity, values exceeding 1.0 indicates a state of anerobic metabolism and is correlated with buildup of lactic acid. Additionally, each individual cage is outfitted with a laser grid allowing for real-time monitoring of ambulatory activity determined by beam breaks created by the animal within the grid. Analysis of data from the CLAMS system was performed using the CalR2 software (Mina et al., 2018).

### Muscle dissection and *ex vivo* assessments

Experimental mice were anesthetized with isoflurane (5% to induce anesthesia; 1.5-2% to maintain) prior to the dissection of extensor digitorum longus (EDL) and soleus muscles. Isometric contractile properties were examined in the muscles *ex vivo*, using established laboratory methodology (Pistilli et al., 2011, 2014). Briefly, nylon sutures were tied to the proximal and distal tendons of the muscles. Muscles were transferred independently to an oxygenated muscle stimulation bath containing Ringer’s solution (100 mM NaCl, 4.7 mM KCl, 3.4 mM CaCl_2_, 1.2 mM KH_2_PO_4_, 1.2 mM MgSO_4_, 25 mM HEPES, and 5.5 mM D-glucose) that was maintained at 22 °C. Muscle stimulation was performed using a commercially available muscle physiology system (Aurora Scientific, Ontario, CA). Muscle length was gradually increased to obtain the maximal twitch force response; this muscle length was recorded as optimal length (L_o_). Muscle contractile parameters obtained from maximal isometric twitch contractions included peak isometric twitch force, contraction time (CT), ½ relaxation time (½ RT), rate of force development (RFD), and rate of relaxation (RR).

With the muscle set at L_o_, muscles were stimulated with 500ms tetanic trains at increasing stimulation frequencies (i.e., 5, 10, 25, 50, 80, 100, 120, and 150 Hz) to establish the force-frequency relationship. Each contraction was followed by a 2-minute rest. Absolute isometric tetanic force was recorded at each stimulation frequency; nonlinear regression was used to generate a sigmoid curve relating this muscle force (*P*) to stimulation frequency (*f*), using the following equation: (Kiriaev et al., 2021).


$$P=P_{min}\;+\;\frac{P_{max}\;-\;P_{min}}{1\;+\;{\left(\frac{K_{f}}{f}\right)}^{h}}$$

The following parameters were obtained from the force-frequency curve: minimum force (*P*_min_), maximum force (*P*_max_), half-frequency (*K*_f_), and the Hill Coefficient (*h*). *K*_f_ is defined as the frequency at which the developed force is the midpoint between *P*_min_ and *P*_max_, where *h* is defined as the slope of the force-frequency sigmoidal curve (Kiriaev et al., 2021). Maximal isometric tetanic force was obtained from the force-frequency curve.

Muscle fatigue was analyzed using repeated 40 Hz tetanic trains that occurred once per second and lasted 330ms, for a total of 6 minutes (Pistilli et al., 2011). Muscle cross-sectional area (CSA) was calculated by dividing the muscle mass by the product of the muscle density coefficient (1.06 g. cm^3^), muscle L_o_, and the fiber length coefficient (EDL: 0.45); this CSA value was used to calculate muscle specific force (i.e., force mN. muscle CSA^–1^) (Brooks and Faulkner, 1988; Lynch et al., 2001).

### RNA-seq

EDL muscles were dissected from SJL mice 14 and 40 days following EAE induction, then homogenized with TissueRuptor Disposable Probes (Qiagen #990890). RNA was then purified using the RNeasy Fibrous Tissue Mini Kit (Qiagen #74704) according to the manufacture protocol. >1μg of RNA from each sample was sent to Admera Health (South Plainfield, NJ) for library construction and sequencing. Isolated RNA quality was assessed by High Sensitivity RNA TapeStation (Aligent Technologies) for RIN values >7 for all samples. Isolated RNA was quantified by Infinite F Nano+ 200 Pro Tecan (Tecan, Switzerland). Libraries were constructed using NEBNext Poly(A) mRNA Magnetic Isolation Module and NEBNext Ultra II Directional RNA Library Prep Kit for Illumina (New England BioLabs). Final library quantities were assessed by Qubit 2.0 (ThermoFisher) and quality was assessed by High Sensitivity D1000 TapeStation ScreenTape (Aligent Technologies). Final library size was ~435bp with an insert size of ~305bp. Equimolar pooling of libraries was performed based on QC values and sequenced on Illumina NovaSeqX Plus 10B (Illumina) with a read length configuration of 150 PE for 40M PE reads (20M in each direction).

RNA-Seq data analysis was performed following protocols established in our previous work (Dziadowicz et al., 2022). Briefly, paired-end reads were aligned to the mouse reference genome (mm10) using Subread v2.0.1 (Liao et al., 2013). Gene-level counts were obtained by summarizing reads aligned to their RefSeq transcripts with featureCounts (Liao et al., 2014). Gene expression levels were quantified as RPKM (reads per kilobase of exon model per million mapped reads) (Mortazavi et al., 2008). Principal component analysis (PCA) was conducted in R. Gene Set Enrichment Analysis (GSEA) (Subramanian et al., 2005) was conducted to infer functional relevance by assessing the significance on the preferential upregulation of predefined reference gene sets in one condition over another using GSEA v4.0.2, with reference gene sets including the MSigDB hallmark collection and Gene Ontology Biological Processes (GOBP). Heatmaps of gene expression patterns were generated using MeV software (Howe et al., 2011). Differentially expressed genes were identified by EdgeR (Robinson et al., 2010) using the following thresholds: FC > 1.5, FDR < 0.05, and expressed in at least one of the compared conditions (RPKM > 3).

### Isolation of mitochondria

EDL and soleus muscles were placed in ATP Medium (100 mM KCl, 10 mM Tris-Base, 5 mM MgSO_4_, 1 mM EDTA, 1mM ATP, 0.5% BSA, pH 7.4) immediately after resected and placed on ice until all samples are collected. The samples were minced in 1 ml of Proteinase Medium (ATP Medium + 2 mg/ml proteinase, type XXIV (Sigma-Aldrich P8038)), then incubated for 10 min on ice with gentle shaking. After incubation, 1 ml ATP Medium was added to inhibit the protease activity. The tissues were homogenized with a Teflon homogenizer with 5 strokes in 2 ml ATP Medium. The homogenate was spun down at 1,600 rpm for 5 min. The supernatant was further spun down at 6,000 rpm for 10 min. The pellet was resuspended in 2 ml KCl Medium (100 mM KCl, 10 mM Tris-Base, 5 mM MgSO_4_, 1 mM EDTA, pH 7.4) and centrifuged at 6,800 rpm for 10 min. Finally, pellet containing isolated mitochondria was resuspended in 100 μl MAS Buffer (220 mM Mannitol, 10 mM Sucrose, 10 mM KH_2_PO_4_, 5 mM MgCl_2_, 2 mM HEPES, 1 mM EGTA, 0.2% fatty acid-free BSA). Protein concentration was determined by Bradford Protein Assay. The samples were diluted to 1 μg/μl for the Seahorse Mitochondria Stress Test.

### Seahorse mitochondria stress test

The Seahorse XF Pro platform (Agilent Technologies) was used to evaluate mitochondrial function by measuring oxygen consumption rate (OCR). One day before the assay, an XF Pro plate was hydrated with 175 μl of calibrant solution per well, sealed, and incubated at 37 °C overnight. On the day of the assay, the assay substrate (100 mM pyruvate and 20 mM malate) and four respiratory modulators (port A: 40 mM ADP solution, port B: 32 μM oligomycin, port C: 40 μM FCCP, port D: 40 μM rotenone and 40 μM antimycin A) were prepared. All were prepared in 10x concentration. The machine was calibrated 1 h prior to the beginning of the assay. To obtain the initial basal reading and the reading after injection of the respiratory modulators, the protocol was set as follow: 2 cycles of 30 sec mix, 30 sec wait, and 2 min measure at 37 °C. In each well, 2.5 μg of isolated mitochondria was loaded, and 12 technical replicates were included for each sample. Each well was then received 20 μl of pyruvate + malate substrate and brought up to 50 μl. After centrifugation at 1,900 x g for 20 min, the wells were brought up to 180 μl and the plate were incubated at 37 °C for 10 min. Seahorse Mitochondria Stress Test was performed using Seahorse XF Pro Analyzer. Data were collected using Seahorse Wave Pro software.

### Treadmill fatigue test

To assess for fatigue-like behavior phenotypes in EAE and control animals, treadmill fatigue testing was performed. The test was started 45 days following induction of disease and exclusion criteria included any animals that still exhibited physical disability due to disease and lasting functional motor deficits such as ataxia. A small number of animals refused to participate in the treadmill acclimation regimen and were also excluded from the study. Treadmill regimen and parameters were adopted from Dougherty et al (Dougherty et al., 2016). An Exer-6M Treadmill with a Simplex Controller (Columbus Instruments) was used to perform the study. Speed parameters were measured in meters per minute (m/min) with time and distance readouts in total seconds spent running and total distance in meters. The apparatus was also equipped with a shock grid to promote animal conditioning during training. The grid was set with shocks at 1Hz and 1mA. On the first day of training, animals were first allowed to briefly explore their individual lane of the treadmill. The treadmill was then set to 1 m/min and care was taken to ensure all animals could walk on the device. Intervention with light tail tapping was used to promote this as needed. Speed was slowly increased to 8 m/min gradually at the rate of 1 m/min every minute. Upon reaching 8 m/min a timer was started and speed was increased to 9 m/min after 5 minutes, 10 m/min at 7 minutes, and finally stopped at 10 minutes. Mice were allowed to briefly explore one last time before returning to standard housing. The parameters for days 2 and 3 of training are as followed: Speed was gradually adjusted up to 10 m/min and a timer was started. Treadmill speed was increased to 11 m/min at 5 minutes, 12 m/min at 10 minutes, and stopped at 15 minutes. On day 4 the fatigue test was carried out. Fatigue-like behavior was determined by animals that remained within 1 body length of the shock grid for 5 consecutive seconds. Animals that performed to failure and remained in the shock grid were deemed as fatigued. Treadmill speed was first brought up to 8 m/min and allowed to “warmup” for 5 minutes. The speed was then raised to 10 m/min and a timer was started. Every 10 minutes the speed of the treadmill was increased at a rate of 2 m/min. The raw readouts that were collected included the total distance ran in meters as well as the total time spent running in seconds.

### Statistical analysis

Statistical analyses were performed using SAS or GraphPad Prism. Specific statistical methods used in each experiment were described in the Figure Legends. For some analysis, AUC was conducted using total AUC setting a baseline parameter to 0. Alpha was set to 0.05 unless otherwise specified, such as when using Tukey-Kramer adjustments for multiple comparisons.

## Results

### Mild RRMS model of EAE to study MS fatigue

**Figure 1:**
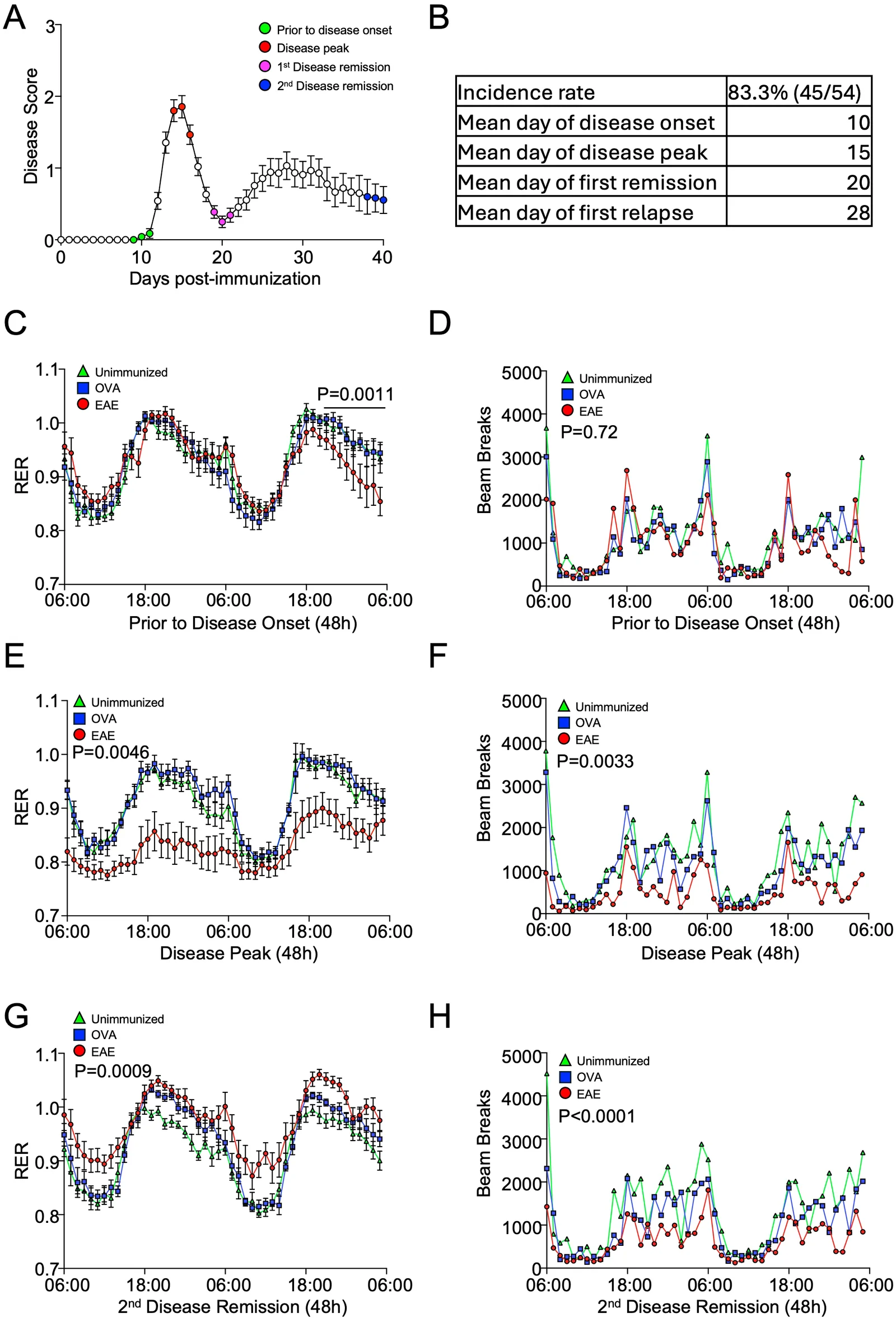
Respiratory Exchange Ratio (RER) dissociates with physical activities of the mice during chronic remission phase. **(A)** Average diseases score of SJL mice immunized with 100 μg [Ser^140^]-PLP_139–151_ emulsified in CFA. Mice were evaluated for 40 days. **(B)** EAE incidence rate, mean days of disease onset, peak, first remission, and first relapse are shown. n = 19. **(C-H)** RER **(C, E, G)** and Locomotion activities **(D, F, H)** of the unimmunized mice, OVA-immunized mice, and EAE-induced mice were monitored using CLAMS. Shown are data extracted 48 h prior to disease onset **(C, D)**, during the peak of disease **(E, F)**, and during the 2^nd^ disease remission phase **(G, H)**. Statistical analysis was performed by comparing area under the curve (AUC) value of each group using ANOVA with Tukey-Kramer adjustment. In C, the last 10 data points of each group were compared using linear mixed modeling. P-values shown are comparison between the unimmunized group and the EAE-induced group. There was no statistical difference between the unimmunized group and the OVA-immunized group in **(C-H)**. n = 6–7 per group.

As most of the MS patients experience fatigue during the early stage of the disease without significant physical disability (i.e., relapse-remitting MS), we determined the feasibility of using a mild RRMS model of EAE to study chronic MS fatigue. We immunized female SJL mice with proteolipid protein 139–151 peptide (PLP_139-151_) emulsified in Complete Freund’s Adjuvant (CFA) without injecting pertussis toxin (PLP-CFA). The mice started to show typical EAE symptoms (see methods section) on day 12. Without pertussis toxin injection, the mice developed only mild physical disability at the peak of the disease (day 14-16), with most demonstrating one hind limb dragging (i.e., score = 2). The mice then rapidly entered the first remission (day 19-21) after the peak of disease, followed by a relapse on day 28, then entered a second remission (day 38-40) (**Figures 1A, B**). Most did not show signs of physical disabilities during remissions, which allowed us to evaluate their physical activities and fatigue levels without physical disability concerns. In addition, some mice were immunized with ovalbumin (OVA) emulsified in CFA (OVA-CFA) to distinguish between the neuroinflammatory mediated effect and the general immune activation caused by CFA. Using this model, we investigated mouse whole-body metabolism and physical properties of the skeletal muscles prior to EAE onset (day 9-11), during the peak of EAE (day 14-16), and during the chronic remission phase (day 38-40).

### Respiratory Exchange Ratio (RER) dissociates with physical activities of the mice during chronic remission phase

We used the Comprehensive Lab Animal Monitoring System (CLAMS) to assess whole-body metabolic changes of the mice over the course of EAE. The respiratory exchange ratio (RER) was calculated by CO_2_ production relative to O_2_ consumption of each mouse, as indicative of the energy source utilized for cellular respiration. In addition, locomotion activities were monitored by infra-red beam sensors. Forty-eight hours prior to the disease onset (day 9-11), RER of all groups (unimmunized, OVA-CFA, EAE) were not significantly different and well correlated with the locomotion activities of the mice. RER raised from 0.8 in the morning to ~1 in the evening indicating the switch of energy source from fat to carbohydrates when the locomotion activities increased (**Figures 1C, D**). Interestingly, RER of the EAE-induced mice dropped in the last 10 hours on day 11, suggesting that metabolic dysregulation began before disease symptoms appeared (**Figure 1C**). During the peak of disease (day 14-16), both RER and locomotion activities were significantly reduced as expected, but the OVA-immunized mice were not affected, suggesting that metabolic dysregulation in this phase was caused by CNS demyelination-mediated disability but not the general immune activation by CFA (**Figures 1E, F**). During the chronic remission phase (day 38-40), even though the EAE-induced mice did not show signs of physical disability, their locomotion activities remained reduced (**Figure 1H**). However, in contrast to the peak of disease, RER of the EAE-induced mice was significantly increased as compared to the unimmunized and OVA-immunized mice (**Figure 1G**), suggesting a potential reduction of mitochondrional capacity. Thus, our data show a dissociation between locomotion activities and whole-body metabolism of the mice during the chronic remission phase of EAE.

### EAE decreases fatigue resistance of the fast extensor digitorum longus (EDL) muscle

**Figure 2:**
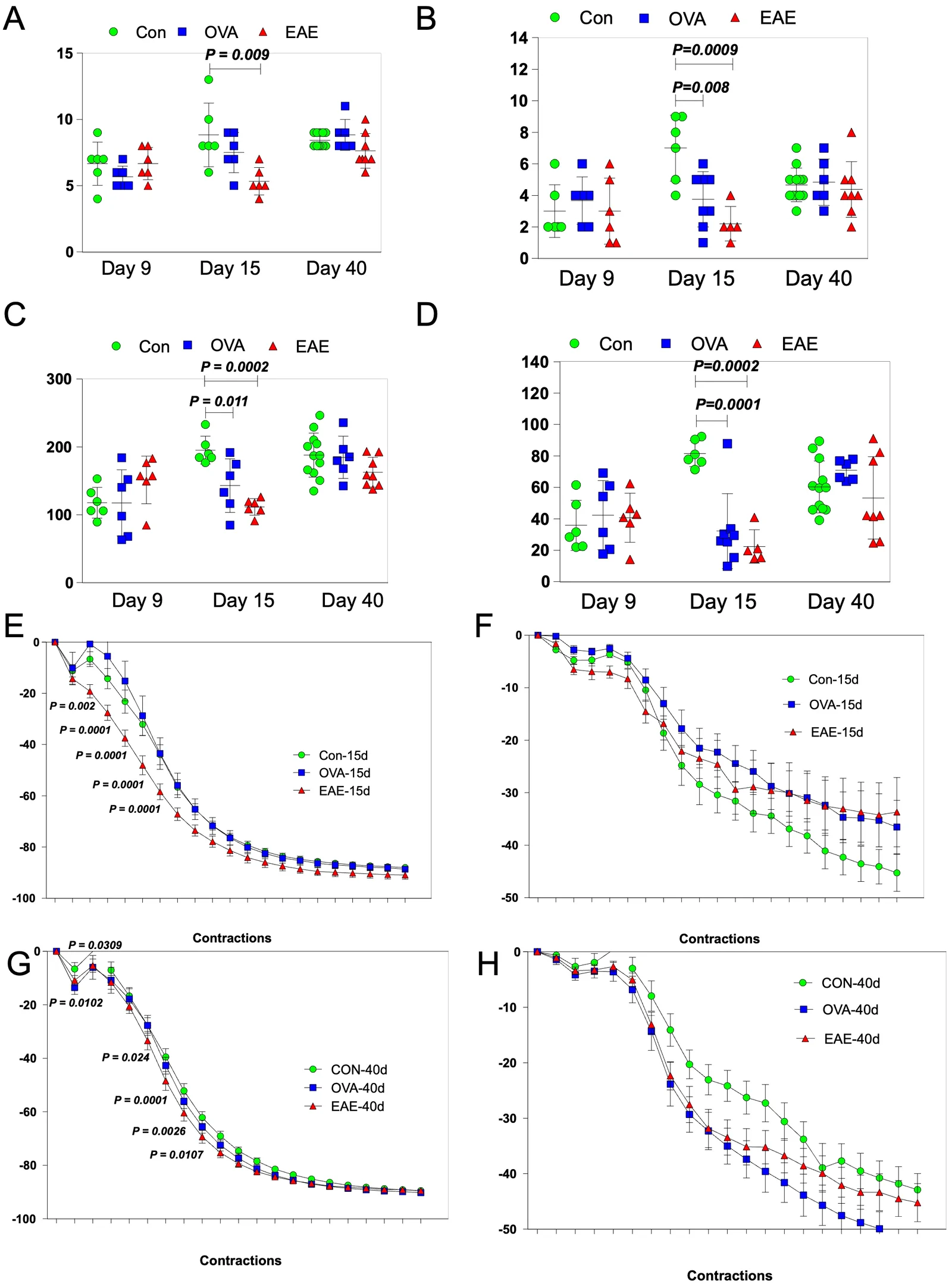
EAE decreases fatigue resistance of the fast EDL muscle. EDL and soleus muscles were dissected from SJL mice on day 9, 15, and 40 following EAE induction (EAE), OVA immunization (OVA), or from mice without immunization (Con). Muscle mass **(A, B)**, maximum force output (tetanus) **(C, D)**, and fatigue index **(E-H)** were measured. Statistical analysis was performed using a one-way ANOVA with Bonferroni *post-hoc* adjustment to compare group means for each day. Day 9: n = 6 per group; Day 15: n = 6 per group; Day 40: n = 12 for control, n = 6 for OVA and n = 8 for EAE.

Next, we asked whether EAE induction directly impacts the force output and fatigue properties of the fast extensor digitorum longus (EDL) and slow soleus muscles. Dissected muscles were stimulated *ex vivo*, representing a model to study the CNS-independent impact to the skeletal muscles. Before disease onset, muscle mass and maximal force output (tetanus) were comparable in the unimmunized, OVA-immunized, and EAE-induced groups. Intriguingly, two weeks after immunization (day 15), muscle mass and force output were significantly reduced in both OVA-immunized and EAE-induced mice as compared to the unimmunized group, suggesting that immunization with a CNS-unrelated antigen in CFA also affects skeletal muscles (**Figures 2A–D**). However, EDL muscle from EAE-induced mice but not OVA-immunized mice showed a fatigue phenotype compared to unimmunized mice (**Figure 2E**). The fatigue phenotype was not observed in soleus muscles, which are relatively fatigue-resistant due to a higher number of mitochondria (**Figure 2F**). Forty days after immunization, muscle mass and force output in both OVA-immunized and EAE-induced groups mostly returned to the level of unimmunized mice (**Figures 2A–D**). However, EDL muscles continued to show a fatigue phenotype, albeit to a lesser extent (**Figure 2G**). Overall, our data show that immunization with either CNS-specific or CNS-non-specific antigens emulsified in CFA reduced muscle mass and force output of both EDL and soleus muscles, but only immunization with a CNS-specific antigen caused EDL muscle fatigue (all data were summarized in **Table 1**).

**Table 1:** Muscle isometric twitch contractile properties.

EDL muscle					SOL muscle				
Day 9	Control	OVA	EAE	p-value	Day 9	Control	OVA	EAE	p-value
Twitch (mN)	26.8 ± 8.7	25.6 ± 8.8	29.7 ± 10.1	0.731	Twitch (mN)	6.0 ± 2.0	5.9 ± 2.4	5.8 ± 1.7	0.986
Twitch CT	20.0 ± 6.3	20.0 ± 8.9	23.3 ± 8.2	0.706	Twitch CT	70.0 ± 8.9	50.0 ± 0.0	55.0 ± 12.2	0.003
Twitch ½ RT	33.3 ± 5.2	30.0 ± 6.3	33.3 ± 5.2	0.506	Twitch ½ RT	96.6 ± 15.1	85.0 ± 15.2	93.3 ± 17.5	0.316
Day 15					Day15				
Twitch (mN)	36.0 ± 5.1	29.5 ± 10.8	30.0 ± 4.2	0.266	Twitch (mN)	9.6 ± 2.0	4.2 ± 2.1	4.0 ± 0.9	P<0.0001
Twitch CT	16.6 ± 5.2	18.3 ± 7.5	21.6 ± 7.5	0.454	Twitch CT	58.3 ± 7.5	60.0 ± 16.0	60.0 ± 12.2	0.967
Twitch ½ RT ^a,b,c^	23.3 ± 5.2	30.0 ± 6.3	36.6 ± 5.1	P<0.001	Twitch ½ RT ^a,b,c^	68.3 ± 7.5	71.5 ± 13.6	96.0 ± 13.4	0.003
Day 40					Day 40				
Twitch (mN)	36.6 ± 8.3	36.7 ± 10.1	31.7 ± 5.0	0.362	Twitch (mN)	6.4 ± 1.9	9.3 ± 0.7	8.4 ± 4.6	0.121
Twitch CT	20.0 ± 4.3	25.0 ± 22.6	10.0 ± 5.3	0.454	Twitch CT	54.1 ± 13.1	61.6 ± 7.5	65.0 ± 10.7	0.117
Twitch ½ RT	26.6 ± 4.9	25.0 ± 5.5	26.2 ± 5.2	0.809	Twitch ½ RT	68.3 ± 10.3	73.3 ± 10.3	85.0 ± 11.9	0.009

^a^Con vs OVA, ^b^CON vs EAE; ^c^OVA vs EAE.

mN, millinewton; CT, contraction time; ½ RT, half relaxation time.

### EDL muscle shows distinct gene signature in active phase and remission phase of EAE

**Figure 3:**
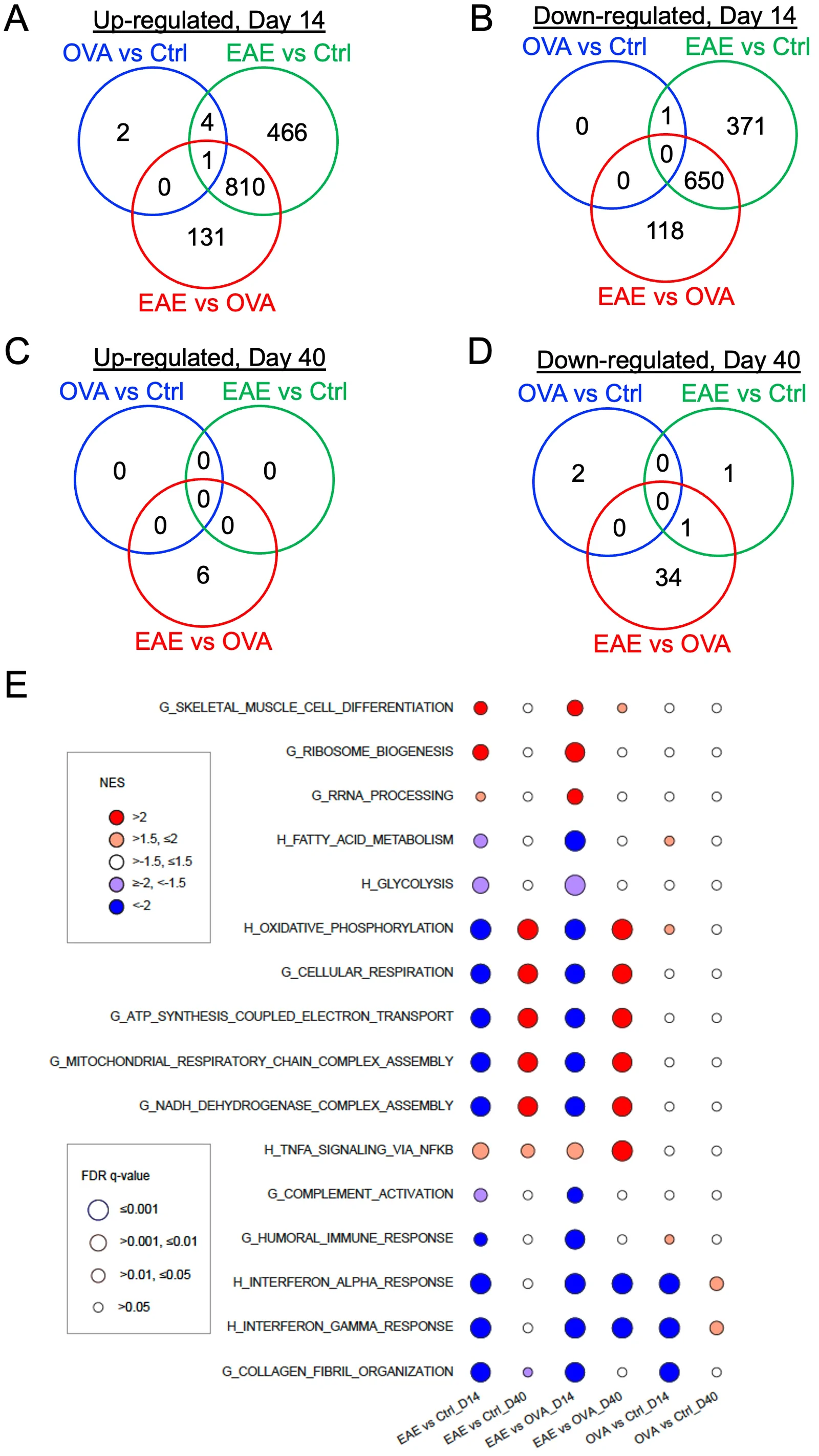
EDL muscle shows distinct gene signature in active phase and remission phase of EAE. EDL muscles were dissected from SJL mice following EAE induction (EAE), OVA immunization (OVA), or from mice without immunization (Ctrl). Gene expression was determined by RNA-Seq. **(A-D)** Venn diagrams showing the number of up-regulated and down-regulated genes (fold change > 1.5; FDR < 0.05) in EDL muscles isolated 14 days **(A, B)** and 40 days **(C, D)** post-immunization. **(E)** GSEA predicted signaling pathways that were induced (NES > 1.5) or suppressed (NES < -1.5) in EAE group relative to Ctrl group or OVA group, and in OVA group relative to Ctrl group, on day 14 (D14) and day 40 (D40). n = 5 per group. NES, Normalized Enrichment Score.

**Figure 4:**
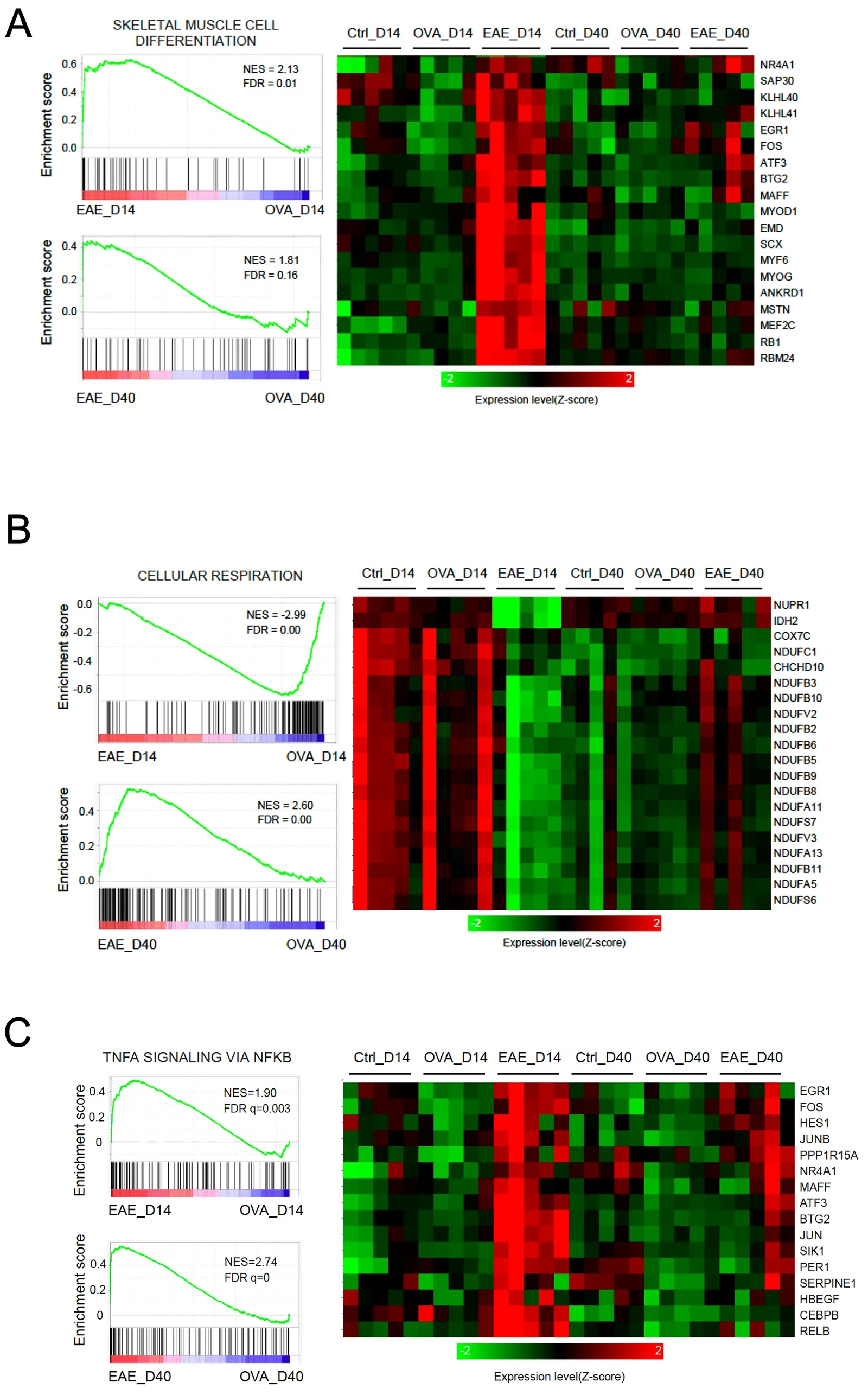
EDL muscles were dissected from SJL mice following EAE induction (EAE), OVA immunization (OVA), or from mice without immunization (Ctrl). Gene expression was determined by RNA-Seq. GESA was performed as described in **Figure 3**. (Left) Shown are graphical presentation of GSEA results of signaling pathways related to skeletal muscle cell differentiation **(A)**, cellular respiration **(B)**, and TNF-α signaling **(C)**. (Right) Heatmaps showing expression of representative genes categorized within the corresponding pathway. n = 5 per group. NES, Normalized Enrichment Score.

Given that EAE induction caused physiological fatigue in the EDL muscle, we evaluated changes of cellular metabolism-related molecular pathways in the EDL muscle on day 14 (peak) and 40 (chronic remission) following EAE induction using RNA-Seq. On day 14, EDL muscle from EAE-induced mice showed distinct gene expression as compared to unimmunized and OVA-immunized groups (**Figures 3A, B**, **Supplementary Figure 1A**). Surprisingly, even though immunization with OVA significantly reduced EDL muscle mass and force output, it minimally impacted gene expression in the EDL muscle (**Figures 3A, B**, blue circles; **Supplementary Figure 1A**). We then used Gene Set Enrichment Analysis (GSEA) to determine whether overall gene expression change within specific molecular pathways correlated with EDL muscle phenotypes observed in our *ex vivo* studies. We found that genes involved in skeletal muscle cell differentiation, ribosome biogenesis, and rRNA processing were upregulated, possibly in response to the rapid EDL muscle deconditioning during the peak of disease and served as a compensatory mechanism (**Figure 3E**, top three rows). In addition, genes in metabolic pathways (fatty acid metabolism, glycolysis, oxidative phosphorylation, cellular respiration, ATP synthesis, etc.) were significantly downregulated (**Figure 3E**, middle seven rows). Interestingly, several immune related pathways, including complement activation, humoral immune response, and interferon alpha and gamma responses, were downregulated, which might correlate with the metabolic dysregulation during the peak of EAE (**Figure 3E**, bottom five rows). On day 40, the number of differentially expressed genes (fold-change > 1.5 and FDR < 0.05) were substantially reduced (**Figures 3C, D**). However, principal component analysis (PCA) showed that gene expression in the EAE-induced group was still separated from the unimmunized and OVA-immunized groups (**Supplementary Figure 1B**). In GSEA, the overall skeletal muscle cell differentiation pathway was upregulated compared to the OVA-immunized group (**Figure 3E**, top and **Figure 4A**). Intriguingly, genes linked to metabolic pathways such as cellular respiration were upregulated (**Figure 3E**, middle and **Figure 4B**). This observation is in support of our whole-body metabolic data in which RER was increased but physical activities of the mice were reduced during the chronic remission phase of EAE (**Figures 1G, H**). Interestingly, the TNF-α signaling pathway was the only immune-related pathway upregulated in EDL muscles in both day 14 and day 40 (**Figure 3E**, bottom and **Figure 4C**), which is known to induce muscle wasting (Reid and Li, 2001). Overall, RNA-Seq analysis suggests that EAE induced metabolic dysregulation of the EDL muscle at the molecular level.

### EAE at the peak phase but not the chronic remission phase reduces mitochondrial oxygen consumption rate (OCR) of EDL muscle

**Figure 5:**
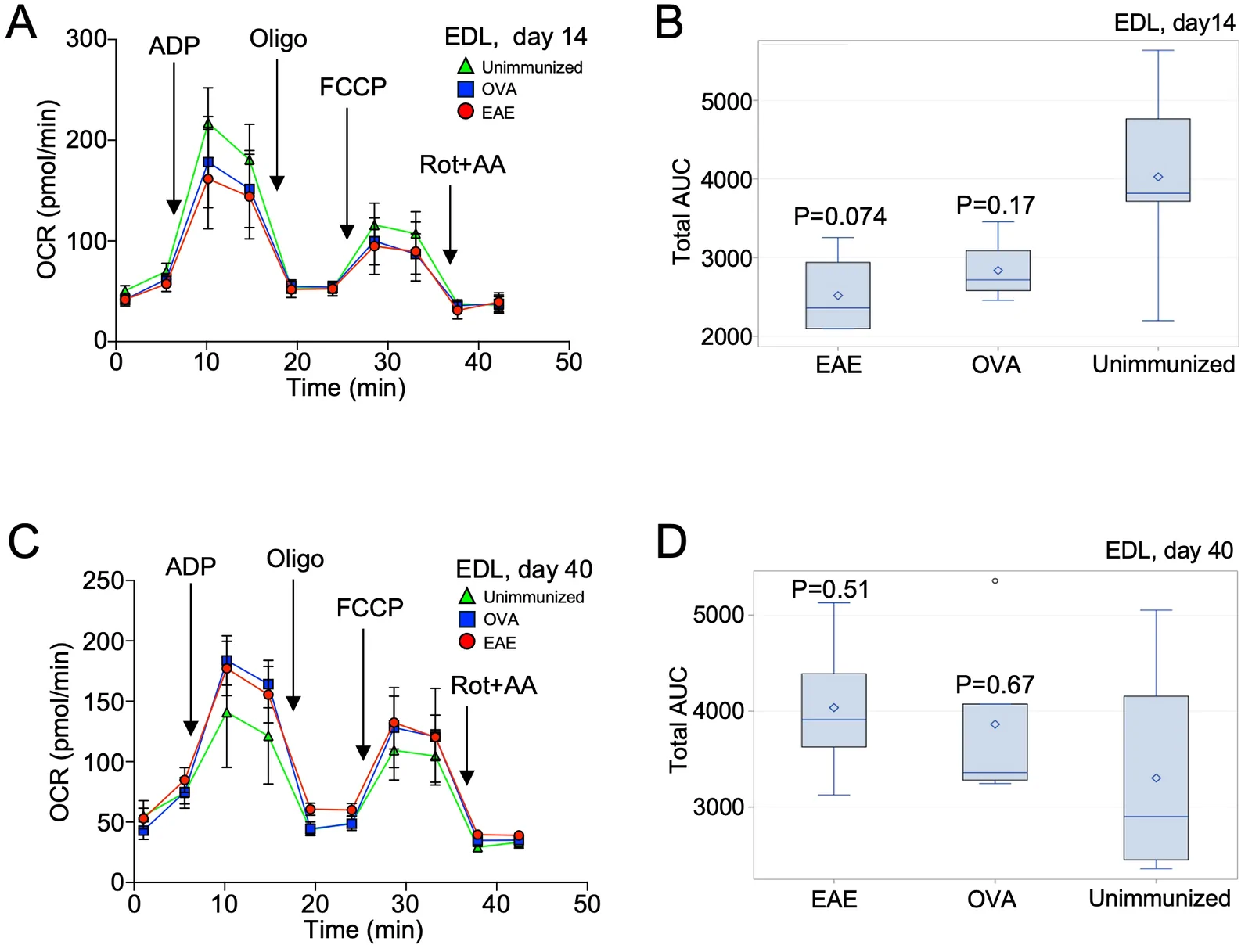
EAE at the peak phase but not the chronic remission phase reduces mitochondrial OCR of EDL muscle. Mitochondria were isolated from EDL muscles 14 days and 40 days following EAE induction, OVA immunization, or from mice without immunization. OCR was determined by Seahorse Mitochondria Stress Test. Combined results from 5 mice on day 14 **(A, B)** and day 40 **(C, D)** are shown. Statistical analysis was performed by comparing area under the curve (AUC) value of each group using ANOVA with Tukey-Kramer adjustment. On day 14, one outlier in EAE group and one outliner in OVA group were excluded during analysis.

We next used Seahorse Mitochondria Stress Test to measure OCR of mitochondria isolated from EDL muscle following EAE induction. On day 14, OCR of EAE group (AUC = 2517.43) was reduced compared with the unimmunized group (AUC = 4027.03) but was not statistically significant (P = 0.074) (**Figures 5A, B**). The moderate reduction of OCR supported data from 14-day RNA-Seq analysis that showed EAE downregulated genes that control cellular respiration (**Figure 3E**). In contrast, on day 40, OCR of EAE group (AUC = 4036.22) was comparable to the unimmunized group (AUC = 3302.30, P = 0.51) (**Figures 5C, D**). These data suggest even though EDL muscle responded by upregulating genes that control cellular respiration during the chronic remission phase of EAE, mitochondrial capacity was not increased.

### EAE-induced mice fatigue quicker in the running test during chronic remission phase

**Figure 6:**
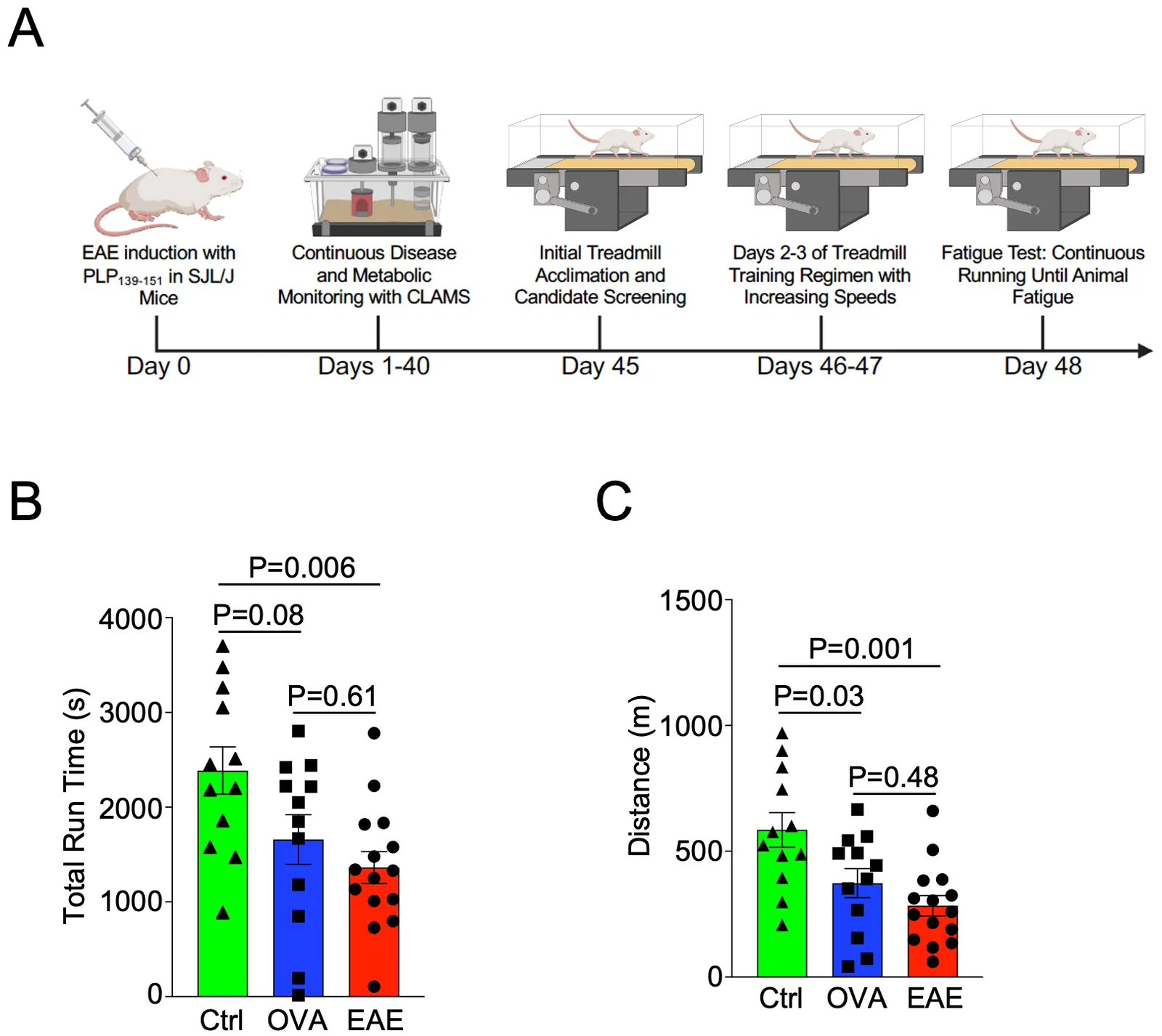
EAE-induced mice fatigue quicker in the running test during chronic remission phase. After 40 days monitoring in CLAMS, EAE-induced mice, OVA-immunized mice, and the unimmunized mice were subjected to treadmill fatigue test. **(A)** A graphic illustration showing the test timeline. The total running time **(B)** and running distance **(C)** were recorded. Statistical analysis was performed by one-way ANOVA and *post-hoc* analysis with Tukey’s test. n = 12 for Ctrl and OVA groups; n = 15 for EAE group. Created in BioRender. Moore, K. (2026) https://BioRender.com/uux5ihz.

Finally, we asked whether the metabolic dysregulation and skeletal muscle phenotypes are correlated with physical fatigue in EAE induced mice. Unimmunized mice, OVA-immunized, and EAE-induced mice that recovered from EAE and did not show any physical disability symptoms at the end of the study were subjected to a treadmill fatigue test 45 days after EAE induction (illustrated in **Figure 6A**). The total run time and distance were then measured. Among 17 EAE induced mice that did not show any physical disability symptoms, two mice refused to run on the treadmill during the training period, indicating severe fatigue. These mice were excluded from the test. We found that both total run time and run distance were significantly decreased in EAE induced mice compared with unimmunized mice (**Figures 6B, C**). Intriguingly, the total run distance of the OVA-immunized mice was also decreased, indicating that CNS-independent immune activation also contributes to physical fatigue.

## Discussion

Although fatigue is the most common symptom experienced by MS patients, the root cause of MS fatigue is not known. In addition, diagnosis of fatigue levels in MS patients is exclusively through self-evaluation using questionnaires such as the Modified Fatigue Impact Scale (MFIS). A key factor to improve our understanding on MS fatigue is to develop an animal model that can use objective parameters to evaluate fatigue levels and investigate the underlying cellular mechanisms. Our studies demonstrate that the RRMS model (SJL-PLP) of EAE is ideal for studying MS fatigue. Unlike the most commonly used EAE model in which C57BL/6 (B6) mice are immunized with myelin oligodendrocyte glycoprotein 35–55 peptide (MOG_35-55_) emulsified in CFA (B6-MOG), SJL-PLP EAE can be induced without pertussis toxin injection, resulting in transient, relatively mild EAE symptoms followed by an asymptomatic remission phase, whereas B6-MOG EAE induces acute demyelination, leading to severe, irreversible physical disabilities. To our knowledge, this is the first report comparing whole-body metabolism, skeletal muscle properties, gene expression, and mitochondrial capacity at different phases of EAE. Importantly, we demonstrated fatigue behavior at the remission phase of SJL-PLP EAE, which is independent of physical disabilities of the mice. A recent study using B6-MOG EAE model demonstrates that EAE induction reduces muscle force and fatigue phenotype, which is consistent with our conclusion (Boesch et al., 2025).

Immune activation is known to trigger metabolic reprogramming to meet a rapid increase of energy demands (Ganeshan and Chawla, 2014). In humans, heightened innate immunity correlates with chronic inflammation, fatigue and post-exertional malaise (Che et al., 2025). One important factor we considered was the effect of heat-killed *Mycobacterium* containing CFA induced activation of innate and adaptive immunity. Therefore, we included a group of SJL mice immunized with OVA emulsified in CFA (OVA-CFA) in our study to distinguish phenotypes caused by CNS-specific versus general immune response to OVA-CFA. OVA-CFA immunization unexpectedly reduced soleus muscle mass and force output of both EDL and soleus muscles 15 days post-immunization. The magnitude of reduction was comparable to the mice at the peak of EAE. In addition, OVA-CFA immunized mice had decreased total running distance in the treadmill fatigue test compared to unimmunized mice. These results indicate that CNS-independent immune activation impacts skeletal muscle strength and is a contributing factor of fatigue. However, mice immunized with OVA-CFA did not show whole-body metabolic alterations. There were also no changes in gene expression and mitochondrial capacity in EDL muscles dissected from OVA-CFA immunized mice. Thus, the effect of CFA is likely unrelated to metabolic dysfunction. In contrast, at the peak of EAE, EDL muscle fatigue correlates with a clear reduction of RER, suggesting that metabolic dysregulation was involved in EAE-induced muscle fatigue. In supporting of this notion, RNA-Seq analysis reveals that genes regulating cellular respiration and ATP synthesis, including the NDUF gene family that encodes subunits of mitochondrial complex I, were downregulated, suggesting EAE causes disruption within EDL muscles to assemble or maintain functionally healthy mitochondria.

Our analysis on the chronic remission phase has provided novel insights into EAE-induced fatigue. During this phase, most mice did not display physical disabilities, yet their locomotive activities were significantly reduced compared to unimmunized or OVA immunized mice, suggesting chronic fatigue. The heightened RER in the EAE-induced mice was due to reduced oxygen consumption rather than carbon dioxide production, possibly indicating a reduction of mitochondria capacity. Although EDL and soleus muscle mass and force output returned to the level observed in unimmunized mice, EDL muscle fatigue persisted. Of note, our muscle fatigue assay was performed *ex vivo* through electrical stimulation, which is independent of neuronal transmission from the CNS. Thus, acute demyelination at peak of EAE causes chronic functional and metabolic dysfunctions of EDL muscles, which could partly explain the fatigue behavior in the treadmill running test. In conclusion, the SJL-PLP EAE model is well suited for investigating the underlying mechanisms of MS fatigue. In MS patients, the first physical attack and relapse may trigger a similar type of chronic metabolic changes, causing long-term fatigue. Future studies will determine the possibility of using whole-body metabolic data and skeletal muscle phenotypes (e.g., through muscle biopsies) as objective parameters to diagnose MS fatigue.

## Data Availability

The raw data supporting the conclusions of this article will be made available by the authors, without undue reservation. The datasets generated for this study are deposited in GEO, under the accession numbers GSE326115.
